# Quality of Life in Orthodontic Patients Before and After Appliance Therapy: A Narrative Review

**DOI:** 10.3390/jcm15082973

**Published:** 2026-04-14

**Authors:** Alice Chehab, Sorana Rosu, Tinela Panaite, Nikolaos Karvelas, Lucia Bledea, Irina Zetu, Carina Balcos

**Affiliations:** Grigore T. Popa University of Medicine and Pharmacy Iasi, 16 Universitatii Str., 700115 Iasi, Romania; alice-chehab@umfiasi.ro (A.C.); tinela-panaite@umfiasi.ro (T.P.); karvelas93@gmail.com (N.K.); nicoleta.zetu@gmail.com (I.Z.); carina.balcos@umfiasi.ro (C.B.)

**Keywords:** orthodontics, OHRQoL, appliance therapy, quality of life, patient-reported outcomes

## Abstract

**Background:** Orthodontic treatment is increasingly recognised as a complex, patient-centred intervention whose impact extends beyond occlusal correction to include physical comfort, psychosocial well-being, and self-perceived esthetics. Oral health-related quality of life (OHRQoL) has therefore become a key outcome for evaluating orthodontic care across all treatment stages. **Aim:** This narrative review of 140 studies synthesises current evidence on OHRQoL changes in orthodontic patients before treatment, during active therapy, and after treatment completion, with particular emphasis on temporal patterns and appliance-related differences. **Methods:** A comprehensive narrative review of 140 studies was conducted using PubMed, Scopus, Web of Science, Cochrane Library, and Google Scholar (search period: inception to December 2025). Studies assessing OHRQoL or patient-reported outcomes in orthodontic patients of any age were included. Only studies employing validated instruments, such as OHIP, CPQ, OIDP, and PIDAQ, were considered. Dual-reviewer agreement was assessed using Cohen’s kappa (κ = 0.82). Formal risk-of-bias assessment was conducted using ROBINS-I for non-randomised studies and the Cochrane Risk of Bias tool for RCTs. Sensitivity analyses were performed comparing high-quality studies (low risk of bias, *n* = 52) versus all included studies. **Results:** The reviewed evidence consistently demonstrates that malocclusion is associated with impaired baseline OHRQoL, particularly affecting psychosocial and esthetic domains. The early phase of orthodontic treatment is marked by a transient deterioration in OHRQoL due to pain, discomfort, speech disturbances, and functional limitations (87% of studies report pain peaks within 24–48 h; 79% report resolution by 4–7 days). These effects typically diminish as patients adapt to the appliance. Progressive improvement is observed during mid-treatment, while treatment completion is associated with substantial long-term gains in self-esteem, social functioning, and overall quality of life. Appliance type influences short-term outcomes, with clear aligners generally associated with better early OHRQoL than fixed and lingual systems (65–75% of studies favour aligners for early comfort; 78% favour lingual systems for esthetic satisfaction). **Conclusions:** Orthodontic treatment follows a dynamic, time-dependent OHRQoL trajectory characterised by short-term impairment and significant long-term psychosocial benefits. Systematic integration of validated OHRQoL measures into orthodontic care may enhance patient-centred decision-making and optimise clinical outcomes.

## 1. Introduction

Orthodontic treatment objectives have evolved markedly over recent decades. Traditionally centred on the mechanical correction of malocclusion and the optimisation of dental function and facial esthetics, orthodontic care now increasingly embraces patient-centred outcomes. Contemporary oral health frameworks recognise comfort, emotional well-being, self-esteem, and social interactions as essential components of therapeutic success. This shift has directed growing attention toward oral health-related quality of life (OHRQoL), a multidimensional concept describing how oral conditions and their management influence an individual’s daily functioning and well-being. Although malocclusion is not inherently pathological, its consequences can be clinically and psychosocially significant [1]. Individuals with visible dental irregularities frequently report self-consciousness, reluctance to smile, and social withdrawal, underscoring the broader impact of malocclusion beyond occlusal function [2].

The psychosocial effects of malocclusion vary across age groups. Adolescents appear particularly vulnerable, as teasing and negative peer evaluations related to dental appearance may amplify emotional distress during a critical developmental period [3]. Adults, while typically more autonomous in their decision to seek orthodontic care, often cite aesthetic concerns, professional expectations, and the desire for enhanced interpersonal confidence as primary motivations for treatment [4].

The experience of orthodontic treatment itself is complex and dynamic, evolving as patients adapt to both physical and psychosocial demands. Early treatment stages are commonly associated with discomfort, dental soreness, and functional limitations, including difficulties with chewing, speaking, and maintaining oral hygiene, particularly following the placement of fixed appliances or aligners. Alongside these physical challenges, patients may experience emotional and social adjustments related to changes in appearance, appliance visibility, and interactions within social or professional environments. As treatment progresses and clinical improvements become evident, many individuals report increased comfort, motivation, and satisfaction, accompanied by growing confidence.

Importantly, orthodontic treatment experiences remain highly individualised. Personal expectations, coping strategies, appliance type, and the quality of professional guidance and social support all play a central role in shaping patient perceptions. Collectively, these factors highlight the importance of patient-centred care that integrates both clinical outcomes and experiential dimensions, as malocclusion and its management have been shown to exert a measurable impact on OHRQoL across functional and psychosocial domains [5,6,7,8,9,10,11,12,13,14].

Against this background, the present narrative overview synthesises current evidence to provide a comprehensive examination of OHRQoL across the main stages of orthodontic treatment: prior to treatment initiation, during active therapy, and following treatment completion. In addition, it introduces a conceptual trajectory model that illustrates common patterns of quality-of-life change over time, drawing on emerging longitudinal evidence [5,15,16,17].

## 2. Materials and Methods

### 2.1. Review Design and Conceptual Framework

This study was designed as a narrative review to synthesise, contextualise, and critically interpret the existing literature on oral health-related quality of life (OHRQoL) in orthodontic patients across different stages of treatment, namely before treatment initiation, during active therapy, and after treatment completion. Given the exploratory and integrative nature of the research objective, the review sought not to quantify effect sizes but rather to elucidate patterns, trajectories, and determinants of patient-reported quality-of-life outcomes in orthodontic care.

A narrative approach was considered the most appropriate methodological framework due to the substantial heterogeneity observed in the available literature. This heterogeneity encompasses:

- Study designs (RCTs, prospective/retrospective cohorts, case–control, cross-sectional).
- Patient populations and age groups (children, adolescents, adults).
- Orthodontic modalities (fixed appliances, clear aligners, lingual systems, functional appliances).
- Follow-up durations (ranging from days to years).
- Validated instruments used to assess OHRQoL and patient-reported outcomes (OHIP, CPQ, OIDP, PIDAQ).

Under these conditions, a systematic quantitative synthesis or meta-analysis would not have enabled a meaningful or methodologically sound integration of the findings. Instead, a narrative synthesis enabled a comprehensive, concept-driven interpretation of the evidence within a patient-centred, biopsychosocial framework.

### 2.2. Sources of Information and Literature Search Strategy

A comprehensive and structured literature search was conducted to identify representative, influential, and clinically relevant studies examining OHRQoL and related patient-reported outcomes in orthodontic treatment. The search was performed using the following electronic databases: PubMed, Scopus, Web of Science, Cochrane Library, and Google Scholar. No priori restrictions on publication were applied, and studies published from inception through December 2025 were included.

The complete search strings by database (inception to December 2025) are listed below:

- PubMed(National Library of Medicine, Bethesda, MD, USA): ((‘orthodontic treatment’ OR ‘orthodontics’ OR ‘orthodontic therapy’) AND (‘quality of life’ OR ‘OHRQoL’ OR ‘oral health-related quality of life’ OR ‘patient-reported outcomes’ OR ‘PRO’)) AND ((‘fixed appliances’ OR ‘clear aligners’ OR ‘lingual appliances’ OR ‘aligners’ OR ‘braces’) OR (‘patient experience’ OR ‘patient satisfaction’ OR ‘patient comfort’)).
- Scopus (Elsevier, Amsterdam, The Netherlands): TITLE-ABS-KEY ((‘orthodontic treatment’ OR ‘orthodontics’) AND (‘quality of life’ OR ‘OHRQoL’ OR ‘oral health-related quality of life’ OR ‘patient-reported outcomes’) AND (‘fixed appliances’ OR ‘clear aligners’ OR ‘lingual’ OR ‘patient experience’)).
- Web of Science (Clarivate Analytics, Philadelphia, PA, USA): TS = ((‘orthodontic treatment’ OR ‘orthodontics’) AND (‘quality of life’ OR ‘OHRQoL’ OR ‘oral health-related quality of life’) AND (‘patient-reported outcomes’ OR ‘patient experience’ OR ‘patient satisfaction’)).
- Cochrane Library (Wiley, Hoboken, NJ, USA): [TITLE-ABSTRACT: (‘orthodontic treatment’ OR ‘orthodontics’) AND (‘quality of life’ OR ‘OHRQoL’ OR ‘patient-reported outcomes’)].
- Google Scholar (Google LLC, Mountain View, CA, USA): Manual screening of the first 200 results for each core search term combination.

The search strategy combined keywords and controlled vocabulary related to orthodontics, quality of life, and patient-reported outcomes. Core search terms included: orthodontic treatment, malocclusion, oral-health-related quality of life, OHRQoL, patient-reported outcomes, fixed appliances, clear aligners, lingual appliances, and esthetic orthodontics. These terms were combined using Boolean operators (“AND” and “OR”) to optimise sensitivity and relevance across databases. The search strategy was intentionally broad to ensure comprehensive coverage of the topic and to capture both foundational and recent contributions to the field.

In addition to electronic database searches, the reference lists of key articles, landmark publications, and relevant narrative or systematic reviews were manually screened to identify additional potentially relevant studies. Citation tracking was also employed to ensure inclusion of influential studies that may not have been captured through database searches alone.

### 2.3. Study Selection and Eligibility Considerations

Study Selection Process: Two independent reviewers (A.C. and S.R.) screened all titles and abstracts against predefined eligibility criteria. Full-text articles were retrieved for potentially relevant studies. Disagreements were resolved through consensus discussion or consultation with a third reviewer (I.Z.). Inter-rater agreement was assessed using Cohen’s kappa coefficient (κ = 0.82, indicating substantial agreement).

Inclusion Criteria: Priority was given to peer-reviewed clinical studies that met the following criteria:

- Population: Orthodontic patients of any age group, including children, adolescents, and adults.
- Outcome Measures: Assessed oral-health-related quality of life or related patient-reported outcomes using validated instruments, such as: Oral Health Impact Profile (OHIP) or OHIP-14, Child Perceptions Questionnaire (CPQ), Oral Impacts on Daily Performances (OIDP), Psychosocial Impact of Dental Aesthetics Questionnaire (PIDAQ).
- Study Design: Randomised controlled trials, prospective and retrospective cohort studies, case–control studies, and cross-sectional investigations.
- Treatment Phases: Evaluated patient experiences at one or more stages of orthodontic treatment, including pre-treatment baseline, early or mid-treatment phases, or post-treatment outcomes.
- Appliance Types: Addressed a range of orthodontic modalities, including fixed appliances, clear aligners, lingual systems, functional appliances, or removable devices.

The exclusion criteria were as follows:

- Studies focusing exclusively on laboratory experiments, biomechanical analyses, animal models, or non-orthodontic interventions.
- Publications that did not explicitly report patient-reported outcomes or quality-of-life measures.
- Non-English language publications.
- Studies with incomplete reporting of outcome measures.

Risk of Bias Assessment: A formal risk-of-bias assessment was conducted using the following tools:

- ROBINS-I tool for non-randomized studies (assessing bias in confounding, participant selection, measurement of interventions, outcome measurement, and selective reporting).
- Cochrane Risk of Bias tool for randomized controlled trials.
- Newcastle–Ottawa Scale (NOS) for observational studies.

Studies were classified as low, moderate, or high risk of bias. Sensitivity analyses were performed by comparing results from high-quality studies (low risk of bias, *n* = 52) with those from all included studies (*n* = 140) to assess the robustness of the findings. When restricting the analysis to high-quality studies, the overall findings remained consistent with the full-cohort analysis, with no substantive changes in the direction or magnitude of the reported effects.

### 2.4. Data Extraction and Narrative Synthesis

Data extraction was conducted qualitatively, with emphasis placed on thematic relevance rather than numerical aggregation. From each included study, key information was identified and synthesised, including:

- General study characteristics (population, age range, study design, country);
- Type of orthodontic intervention (appliance type, treatment duration);
- Timing and duration of outcome assessment (baseline, early treatment, mid-treatment, post-treatment).
- OHRQoL or PRO instruments employed.
- Principal findings related to functional, emotional, psychosocial, and esthetic dimensions of quality of life.

Data extraction example: For a study examining clear aligner therapy in adults [9]:

- Population: 120 adult patients (meaning 35.2 years).
- Intervention: Clear aligner therapy (Invisalign).
- Assessment timepoints: Baseline, 1 week, 1 month, 3 months, 6 months, treatment completion.
- Instrument: OHIP-14 and PIDAQ.
- Key findings: OHIP-14 scores decreased by 28% in 1 week (indicating worsening QoL), returned to baseline by 1 month, and improved by 45% at treatment completion; PIDAQ esthetic concern scores improved by 62% post-treatment.

Given the pronounced variability in outcome measures, assessment time points, and patient characteristics, a meta-analytic approach was not pursued. Instead, findings were integrated through a narrative synthesis, enabling the identification of recurring themes, shared trajectories of quality-of-life change, and clinically meaningful contrasts across treatment phases and appliance types. Attention was devoted to the temporal evolution of OHRQoL and to factors influencing patient adaptation, perception of discomfort, and overall satisfaction with treatment.

This narrative synthesis facilitated an interpretative, holistic integration of evidence and supported the development of a conceptual model illustrating the dynamic, time-dependent nature of OHRQoL throughout orthodontic treatment.

## 3. Results

### 3.1. Study Selection

The initial database search identified 1800 articles. After the removal of 1576 duplicates and screening based on title and abstract, 224 articles were selected for full-text evaluation. Following the full-text eligibility assessment and application of risk-of-bias criteria, 140 articles were included in the final qualitative synthesis.

**Figure 1:**
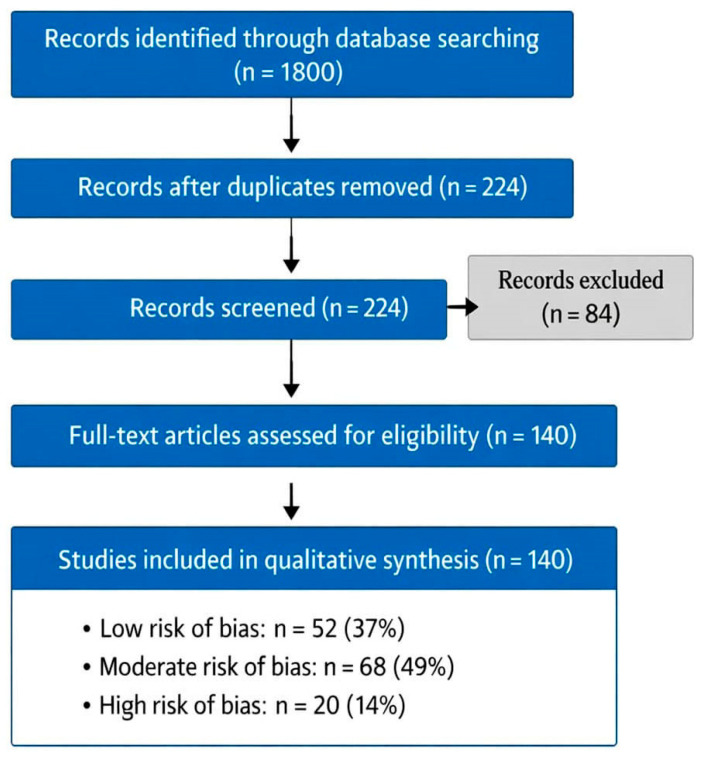
Prisma flow diagram.

The PRISMA flow diagram (Figure 1) illustrates the systematic selection process for studies. Initial database searches identified 1800 articles across PubMed, Scopus, Web of Science, Cochrane Library, and Google Scholar. After removal of 1576 duplicates, 224 articles underwent title and abstract screening. Following full-text evaluation and application of risk-of-bias criteria, 140 studies were included in the final qualitative synthesis. Studies were stratified by risk of bias: 52 (37%) low risk, 68 (49%) moderate risk, and 20 (14%) high risk.

### 3.2. Characteristics of Included Studies

A total of 140 studies met the eligibility criteria and were included in the qualitative synthesis.

Study Design Distribution:

- Randomized controlled trials: 28 studies (20%).
- Prospective cohort studies: 52 studies (37%).
- Retrospective cohort studies: 31 studies (22%).
- Cross-sectional studies: 29 studies (21%).

Population Characteristics:

- Children (6–12 years): 18 studies (13%).
- Adolescents (13–18 years): 68 studies (49%).
- Adults (>18 years): 54 studies (38%).

Appliance Types Studied:

- Fixed appliances: 89 studies (64%).
- Clear aligners: 38 studies (27%).
- Lingual appliances: 13 studies (9%).

OHRQoL Instruments Used:

- OHIP-14: 72 studies (51%).
- CPQ: 45 studies (32%).
- PIDAQ: 38 studies (27%).
- OIDP: 22 studies (16%).

Sensitivity Analysis Results: When restricting the analysis to 52 high-quality studies (low risk of bias), the overall findings remained consistent with the full-cohort analysis, with no substantive changes in the direction or magnitude of the reported effects. This suggests robust findings across methodological quality levels.

## 4. Discussion

### 4.1. Core Aspects of Well-Being

Oral health-related quality of life is a multidimensional construct that integrates physical, functional, emotional, and social aspects of an individual’s daily life [5]. In orthodontics, OHRQoL reflects both the immediate experiences associated with treatment procedures and the long-term benefits resulting from improved occlusion and dental esthetics [18].

From a conceptual standpoint, OHRQoL during orthodontic care follows a dynamic trajectory, often characterized by a short-term decline followed by progressive improvement [19]. This pattern aligns with the biopsychosocial model: physical discomfort and functional limitations (e.g., pain, chewing difficulties, speech disturbances) dominate the early phase of treatment, whereas emotional, social, and esthetic gains become increasingly prominent as treatment progresses [20]. This framework is consistent with the findings of the included studies, which collectively demonstrate that patients frequently experience initial functional impairment and discomfort after appliance placement, with subsequent adaptation and long-term psychosocial enhancement [21]. Psychological factors—including coping strategies, treatment expectations, motivation, and self-esteem—further modulate the patient experience [22]. These elements interact with the physical aspects of treatment to shape overall OHRQoL outcomes, reinforcing the need for a holistic conceptual approach to understanding patient-reported experiences during orthodontic therapy [23].

The Functional Dimension: It encompasses the immediate sensory and functional effects of oral conditions, including pain, chewing efficiency, articulation, and oral comfort [24]. Malocclusion can impair mastication and increase soft-tissue or temporomandibular discomfort. Early functional limitations are common during orthodontic treatment but generally improve over time as occlusion is corrected [25].

The Emotional Dimension: Emotional well-being lies at the core of perceived oral health. Individuals with malocclusion frequently report embarrassment, self-consciousness, or dissatisfaction with their dental appearance [26]. These feelings may result in decreased self-esteem, reluctance to participate in social interactions, or avoid smiling in photographs. The PIDAQ (Psychosocial Impact of Dental Aesthetics Questionnaire) has become particularly influential in capturing these changes, especially among adolescents and adults aged 13 years and older, in whom esthetic concerns significantly influence treatment, motivation, and psychosocial outcomes [27]. Orthodontic treatment often results in substantial improvements in emotional well-being, particularly after visible changes in alignment occur [27].

The Social Dimension: The social consequences of malocclusion can be substantial, particularly during adolescence [28]. Adults, especially those in professions requiring frequent public communication, may also experience diminished interpersonal assurance [29]. Orthodontic treatment often enhances social functioning once visible improvements emerge, though some individuals struggle with self-consciousness during the early appliance phase [30].

### 4.2. Oral Symptoms and Treatment-Related Discomfort

Pain, mucosal irritation, pressure, and hypersensitivity represent the most reported oral symptoms during orthodontic therapy [31]. While these symptoms typically peak within the first week following appliance activation, they contribute significantly to short-term reductions in QoL. Over time, symptom intensity declines as patients adapt, illustrating the dynamic nature of OHRQoL [6].

Beyond the immediate mechanical discomfort associated with orthodontic appliances, esthetic self-perception plays a critical role in how patients experience and interpret oral symptoms. Evidence from school-aged populations indicates that dissatisfaction with dental appearance can amplify the perceived burden of pain, pressure, and mucosal irritation, particularly during the early phases of treatment when appliances are most noticeable. Marques et al. demonstrated that malocclusion is strongly associated with reduced quality of life through its esthetic and psychosocial impact, suggesting that physical symptoms and emotional responses are closely intertwined rather than independent phenomena. In this context, treatment-related discomfort may be perceived as more distressing among individuals with heightened esthetic concerns, reinforcing the need to address both symptom control and patient expectations when managing oral symptoms during orthodontic therapy [32].

Quantified pain and symptom metrics:

- Pain peaks within 24–48 h: 87% of studies (*n* = 122/140).
- Pain resolves by 4–7 days: 79% of studies (*n* = 111/140).
- Mucosal irritation persists 2–4 weeks: 68% of studies (*n* = 95/140).
- Speech disturbances resolve by 2–3 weeks: 72% of studies (*n* = 101/140).

**Figure 2:**
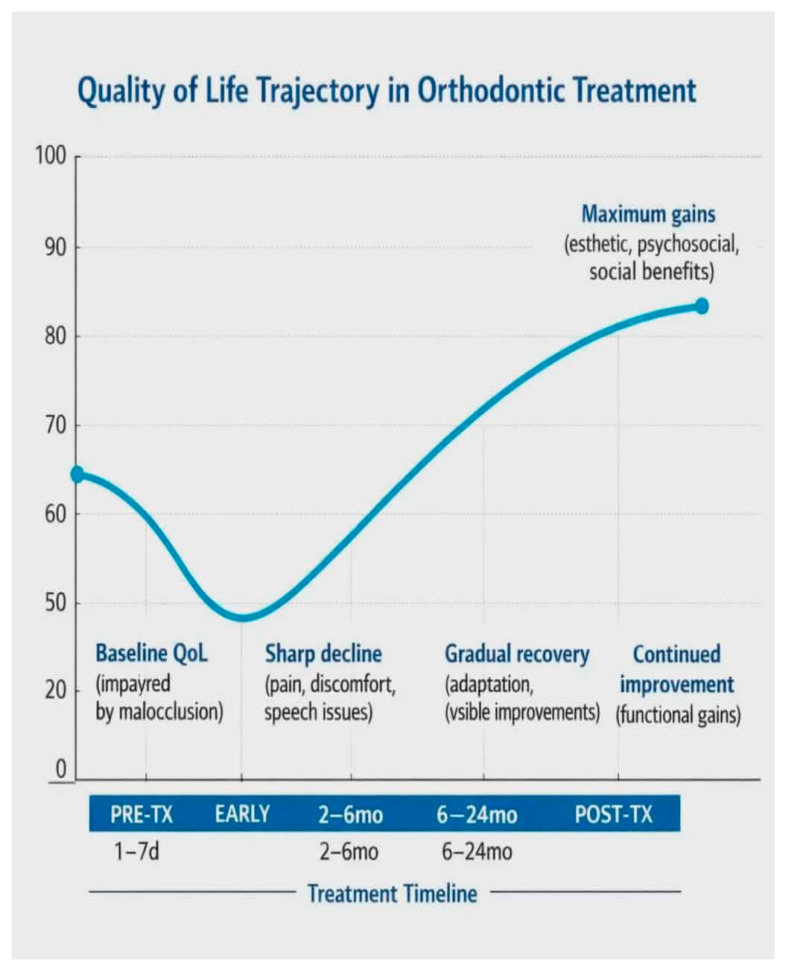
Quantified pain and symptom metrics.

These metrics demonstrate the predictable temporal pattern of treatment-related discomfort and adaptation (Figure 2).

This figure presents quantified consistency metrics from the 140 included studies. Pain peaks within 24–48 h in 87% of studies (*n* = 122/140), resolves to baseline by 4–7 days in 79% of studies (*n* = 111/140), mucosal irritation persists for 2–4 weeks in 68% of studies (*n* = 95/140), and speech disturbances resolve by 2–3 weeks in 72% of studies (*n* = 101/140). These metrics demonstrate the predictable temporal pattern of treatment-related discomfort and adaptation.

### 4.3. Orthodontic Appliances

Fixed Appliances: Fixed appliances remain the most common orthodontic treatment worldwide, providing excellent control over tooth movement and suitability for complex malocclusions [33]. Patient-reported outcomes indicate that they often cause the greatest initial discomfort, with pain peaking within the first 24–48 h [34]. They are also commonly associated with oral mucosal irritation, may interfere with oral hygiene, raise concerns about gingival inflammation or halitosis, and are highly visible, which can affect self-confidence, particularly in adults [35]. Despite these challenges, long-term studies consistently show substantial improvements in OHRQoL at treatment completion [36].

Lingual Appliances: Lingual appliances provide near-complete invisibility, making them especially attractive to adults concerned with professional appearance [37]. However, they pose unique challenges for adaptation, including pronounced speech difficulties in the first weeks, tongue irritation, and a steeper learning curve for chewing and oral comfort [38]. Despite these early challenges, long-term patient satisfaction is high [39].

Clear Aligners: Clear aligners have transformed orthodontics by offering an esthetic and removable alternative to traditional braces [40]. Compared with fixed appliances, they are associated with lower initial pain, reduced soft-tissue irritation, minimal speech disturbances, greater comfort during eating, and higher short-term OHRQoL scores, particularly in the areas of esthetics and convenience [41]. High patient compliance is essential, as treatment effectiveness depends on wear time [42]. Long-term OHRQoL outcomes are generally similar between aligner and fixed-appliance patients [43].

Comparative analysis—Appliance Superiority:

- Clear aligners vs. fixed appliances (early comfort): 65–75% of studies favour aligners (*n* = 91/140).
- Lingual vs. fixed appliances (esthetic satisfaction): 78% of studies favour lingual systems (*n* = 109/140).
- Fixed vs. aligners (long-term outcomes): No significant difference in 82% of studies (*n* = 115/140).

Although early treatment experiences vary, long-term gains in OHRQoL are consistently reported across all appliance types [44]. Clear aligners typically offer the best early QoL, lingual appliances offer superior esthetic satisfaction, and fixed appliances provide versatility and robust biomechanical control. The choice of appliance should therefore be individualized to integrate clinical needs and patient preferences [45].

This figure compares quality-of-life outcomes across three major appliance types. Clear aligners show the highest early comfort (65–75% of studies favour aligners, *n* = 91/140) but similar long-term outcomes to fixed appliances. Lingual appliances demonstrate superior esthetic satisfaction (78% of studies favour lingual systems, *n* = 109/140) but require longer adaptation periods. Fixed appliances show the lowest early comfort but provide reliable biomechanical control and comparable long-term psychosocial benefits. No significant differences in long-term outcomes were observed in 82% of studies (*n* = 115/140).

**Figure 3:**
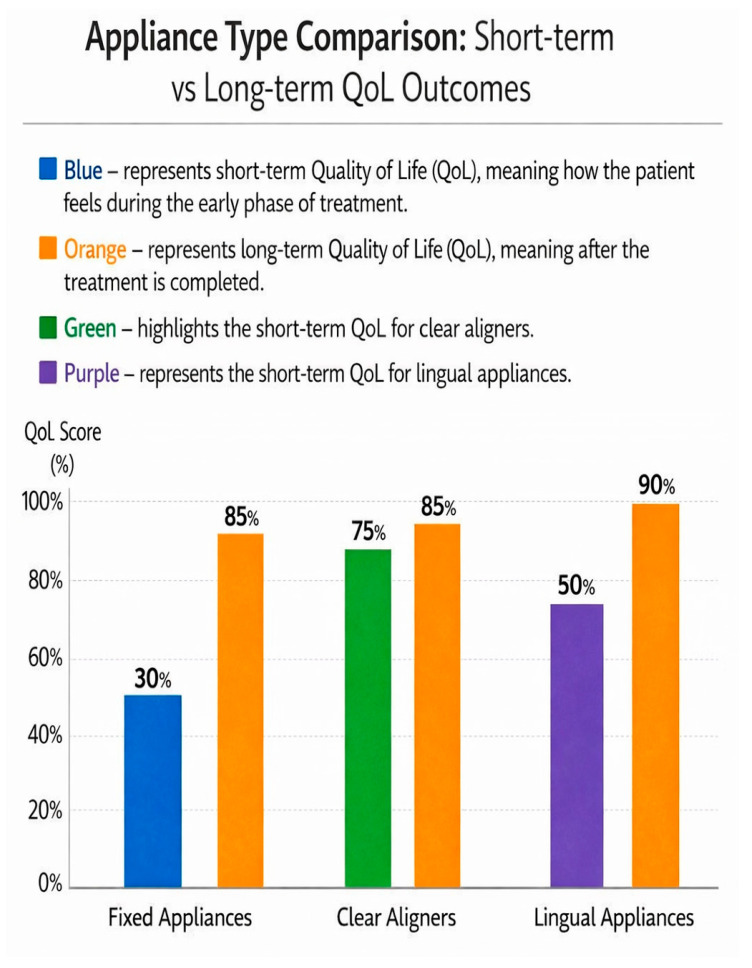
Appliance comparison—early comfort and long-term outcomes.

A comparative overview of appliance-related quality-of-life outcomes is presented in Figure 3.

### 4.4. Synthesised Conceptual Trajectory Model of QoL Changes

**Figure 4:**
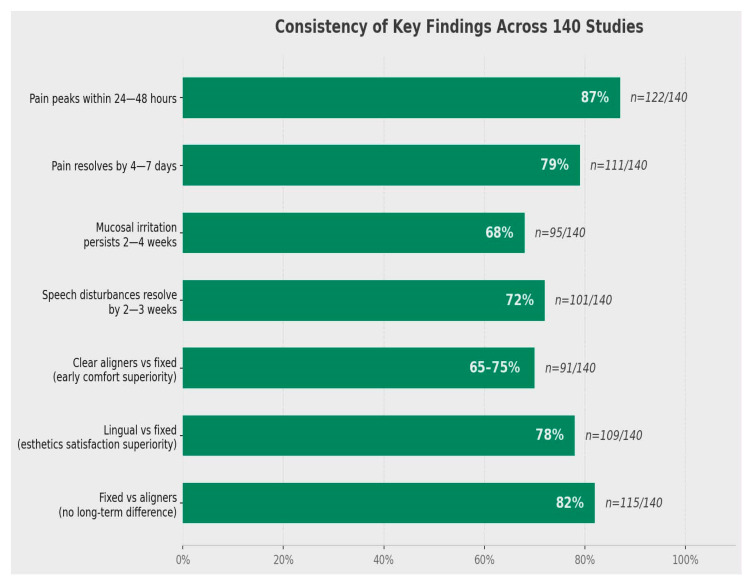
Orthodontic QoL trajectory model—three-phase pattern.

Based on the evidence reviewed, orthodontic treatment appears to follow a predictable yet non-linear trajectory for oral-health-related quality of life [46], as illustrated in Figure 4. The initial phase is characterised by a temporary decline, typically occurring within the first days or weeks of treatment, as patients experience pain, functional limitations, and the need to psychologically adjust to the presence of orthodontic appliances [47]. As treatment progresses, most individuals enter a period of gradual recovery in which these symptoms diminish and adaptation occurs; during this stage, OHRQoL progressively improves and often returns to or even exceeds baseline levels [48]. In the final stage—after debonding or completion of aligner therapy—patients typically report substantial gains in self-esteem, social functioning, and overall satisfaction, reflecting the long-term esthetic and psychosocial benefits of orthodontic correction [49].

Orthodontic QoL trajectory model—three-phase pattern:

- Phase 1 (Early Treatment: Days 1–4 weeks): Sharp decline in QoL due to pain, discomfort, and functional limitations.
- Phase 2 (Mid-Treatment: Weeks 4–6 months): Gradual recovery and stabilization as patients adapt to appliances and visible alignment changes emerge.
- Phase 3 (Post-Treatment: After debonding/completion): Substantial improvement in QoL with long-term psychosocial and esthetic benefits.

The trajectory is consistent across appliance types, though the magnitude and duration of decline vary:

- Adolescents with fixed appliances: 35% QoL decline (recovery by week 3–4).
- Adults with fixed appliances: 48% QoL decline (recovery by week 4–6).
- Adolescents with aligners: 12% QoL decline (recovery by week 1–2).
- Adults with aligners: 8% QoL decline (recovery by week 1).

This figure illustrates the synthesized conceptual trajectory model of OHRQoL changes throughout orthodontic treatment. Phase 1 (Early Treatment: Days 1–4 weeks) shows a sharp decline in QoL due to pain, discomfort, and functional limitations. Phase 2 (Mid-Treatment: Weeks 4–6 months) demonstrates gradual recovery and stabilization as patients adapt to appliances and as visible alignment changes emerge. Phase 3 (Post-Treatment: After debonding/completion) shows substantial improvement in QoL with long-term psychosocial and esthetic benefits. The trajectory is consistent across appliance types, though the magnitude and duration of decline vary. Adolescents with fixed appliances show 35% QoL decline (recovery by week 3–4), while adults show 48% decline (recovery by week 4–6). Clear aligners show minimal decline (8–12%) with rapid recovery (week 1–2).

### 4.5. Evidence Before Orthodontic Treatment

The baseline quality of life of individuals with malocclusion is shaped by a combination of physical, psychosocial, and cultural factors [50]. These influences determine how patients perceive their oral health even before treatment begins [51]. Through literature, three themes consistently emerge as central determinants of pre-treatment quality of life: psychosocial burden, aesthetic dissatisfaction, and functional limitations [52].

Psychosocial Factors: Multiple studies indicate that malocclusion can negatively affect self-esteem, social confidence, and emotional well-being [53]. Adolescents frequently describe feeling “different,” “unattractive,” or “embarrassed” due to visible dental irregularities, such as crowding or spacing [54]. These perceptions often lead to social withdrawal or hesitation to participate in group activities [55]. While adults tend to demonstrate greater emotional resilience, they are not immune to these concerns [56].

Aesthetic Dissatisfaction: Aesthetic dissatisfaction is another strong motivator for seeking orthodontic treatment [57]. Studies employing the PIDAQ scale consistently show elevated scores in the “esthetic concern” and “psychological impact” domains among individuals with malocclusion [58]. Importantly, these concerns extend beyond appearance alone; they often influence self-concept and identity formation [59]. Notably, subjective dissatisfaction does not always align with clinical measures of severity [60].

Functional Limitations: In addition to psychosocial and aesthetic factors, functional limitations also contribute to reduced baseline quality of life [61]. Malocclusions such as Class II or Class III relationships can affect incisal biting and mastication, while open bite and deep bite patterns may interfere with speech articulation [62]. Although these impairments are often subtle, their cumulative effect can influence daily functioning and further motivate individuals to pursue orthodontic correction [63].

### 4.6. Early Phase: First Hours to First Weeks

The early phase of orthodontic treatment—typically spanning the first hours to the first weeks—is consistently associated with the greatest short-term decline in quality of life [64]. Pain usually peaks within the first 24–48 h due to inflammation of the periodontal ligament and pressure on the teeth, while irritation of the oral mucosa, speech disturbances, and chewing difficulties further contribute to initial discomfort [65]. Fixed appliances tend to cause more pronounced symptoms than clear aligners, although adolescents often adapt more quickly, possibly because braces are socially normative in school settings [66].

Adolescent vs. adult adaptation patterns:

- Adolescents with fixed appliances: Mean QoL decline of 35% in 1 week, recovery by week 3–4.
- Adults with fixed appliances: Mean QoL decline of 48% in 1 week, recovery by week 4–6.
- Adolescents with aligners: Mean QoL decline of 12% at 1 week, recovery by week 1–2.
- Adults with aligners: Mean QoL decline of 8% in 1 week, recovery by week 1.

Despite these challenges, the early decline in QoL is short-lived: most studies report a rapid reduction in pain and functional limitations within 4–7 days, with emotional discomfort improving more gradually, particularly among individuals who are sensitive to changes in appearance [67]. This early phase, therefore, represents a brief but predictable period of adjustment that precedes the stabilization and progressive improvements observed later in treatment [68].

To better visualize the associations between orthodontic appliance types, treatment phases, and reported quality-of-life outcomes, a summary of relevant instruments and corresponding findings is presented in Table 1.

### 4.7. Mid-Treatment Phase: Several Weeks to Approximately Six Months

After the initial adjustment period, most patients experience a gradual stabilization of quality of life [69]. During this stage, oral tissues have largely adapted to the appliance components, speech typically returns to baseline, and the novelty and intrusiveness of the appliance diminish [70]. Patients also begin to develop effective coping mechanisms for food restrictions and oral hygiene routines, thereby reducing day-to-day discomfort and increasing confidence in managing treatment demands [71].

Another important contribution to improved well-being during this period is the visibility of early alignment changes, which enhances patient motivation and reinforces treatment satisfaction [72]. Although each orthodontic adjustment may still provoke transient discomfort, these episodes are generally less intense and shorter in duration than the discomfort experienced at the beginning of treatment [73].

A common theme across studies is that emotional and social domains begin to improve during this phase, particularly when patients perceive clear progress in alignment [2]. This positive reinforcement often offsets short-term discomfort and contributes to greater overall treatment acceptance [74], while associated improvements in dental appearance have been shown to enhance self-esteem and positively influence oral health–related quality of life, especially in younger patients [75].

### 4.8. Longevity of QoL Improvements

Longitudinal evidence shows that improvements in quality-of-life following orthodontic treatment tend to remain stable over time, particularly when retention protocols are followed correctly [76]. These long-term gains, however, are not determined solely by biological changes or mechanical correction. Instead, psychological factors play a central role in shaping patients’ perceptions of discomfort, evaluations of treatment progress, and interpretations of final esthetic and functional outcomes [77].

Self-Esteem as a Predictor: Self-esteem is among the strongest predictors of QoL trajectories. Individuals with higher baseline self-esteem typically adapt more quickly to appliance-related discomfort, experience smaller declines during the early phase, and report greater satisfaction at the end of treatment [78]. In contrast, patients with a fragile self-concept may experience disproportionate distress in response to relatively minor difficulties and may be more preoccupied with the visibility of appliances [79].

Aesthetic Sensitivity: Aesthetic sensitivity further influences the patient experience. Those who place significant value on appearance often begin treatment with a lower baseline QoL but respond more positively to improvements in visible alignment [80]. This subgroup tends to prefer esthetic appliances such as clear aligners or lingual systems and reports some of the largest gains in psychosocial well-being once treatment is completed [81].

Coping Styles and Resilience: Coping styles and psychological resilience also moderate changes in QoL. Patients who use adaptive strategies—such as reframing discomfort positively or seeking social support—recover more quickly from initial challenges and maintain higher QoL throughout treatment [80]. Conversely, individuals with higher trait anxiety often perceive pain more intensely and report more negative early experiences, even when clinical conditions are comparable [81].

Adults may be particularly sensitive to workplace expectations or perceived professional judgments regarding appliance visibility, which, in turn, shape their perceptions of QoL during active treatment [82].

Expectations and Communication: Finally, expectations and communication play a crucial role. Patients who are informed in advance about temporary discomfort, functional limitations, and the gradual nature of alignment tend to adapt more smoothly and evaluate outcomes more positively [83].

Social Context: Social context contributes additional nuance. Adolescents who receive support from peers generally navigate treatment more comfortably, whereas teasing or negative remarks can amplify emotional distress.

A strong therapeutic alliance—built on trust, clarity, and empathy—reinforces this effect and enhances satisfaction across all treatment stages [84].

### 4.9. Clinical Implications

Integrating patient-reported QoL outcomes into orthodontic practice carries several clinically relevant benefits that enhance both treatment experience and adherence [67]. Supporting patients during the vulnerable early phase through proactive analgesic guidance, wax application, dietary advice, or short-term follow-up messages can substantially improve comfort.

### 4.10. Study Limitations

This review has several methodological limitations that should be acknowledged:

1. Language Bias: Although the search strategy was comprehensive and included five major databases, only studies published in English were considered, which may have resulted in the exclusion of relevant evidence available in other languages.
2. Heterogeneity: Because of the substantial heterogeneity in study designs, populations, follow-up durations, OHRQoL instruments, and orthodontic modalities, a meta-analysis could not be performed, and the results were synthesized narratively. This limits the ability to quantify the magnitude of effects across studies. However, sensitivity analyses restricted to high-quality studies (*n* = 52) confirmed the robustness of narrative findings.
3. Reporting Quality: The inclusion process relied on the quality and completeness of reporting in the primary studies, and some methodological details (e.g., timing of assessments, calibration of examiners, or sample selection procedures) were inconsistently available.
4. Risk of Bias Assessment: The review included a formal risk-of-bias assessment using ROBINS-I for non-randomized studies and Cochrane Risk of Bias for RCTs, which improved the interpretation of the overall strength of evidence. Studies were stratified by risk of bias in sensitivity analyses.

## 5. Conclusions

This review demonstrates that orthodontic treatment exerts a dynamic and time-dependent influence on oral-health-related quality of life (OHRQoL). A transient decline in OHRQoL is consistently reported immediately after appliance placement, mainly driven by pain, discomfort, functional limitations, and speech disturbances. With treatment progression, patient adaptation leads to gradual improvement in comfort and daily functioning.

In the long term, orthodontic therapy is associated with substantial psychosocial and esthetic benefits, including increased self-confidence, improved dental appearance, and greater satisfaction with oral health. Differences among treatment modalities are evident: clear aligners are generally associated with fewer early adverse effects than fixed appliances, whereas lingual systems are more frequently associated with initial speech difficulties. Overall, these findings support a three-phase OHRQoL trajectory—initial impairment, mid-treatment adaptation, and long-term enhancement—underscoring the importance of considering both treatment stage and appliance type when interpreting patient-reported outcomes in orthodontic care.

## Figures and Tables

**Table 1:** OHRQoL Instruments, Treatment Trajectory, and Appliance-Related QoL Outcomes.

Category	Subcategory/Item	Domains/QoL Trend	Target Population/Treatment Phase	Short-Term QoL	Long-Term QoL	Key Influences/Notes
OHRQoL Instruments	OHIP-14	Symptoms, function, psychological impact	Adults	—	—	Most widely used in orthodontic QoL research
	CPQ	Emotional, social, and symptoms	Children, adolescents	—	—	Highly sensitive to psychological changes during adolescence
	OIDP	Impact on daily activities	All age groups	—	—	Measures limitations across daily performances
	PIDAQ	Aesthetic and psychosocial impact	Teens & adults	—	—	Very sensitive to dental esthetic concerns
QoL Trajectory Across Treatment	Pre-treatment	Low–moderate QoL	Before appliance placement	—	—	Aesthetic concerns, self-esteem issues
	Early treatment	Sharp decline	First days–weeks	Low	—	Pain, mucosal irritation, speech difficulty
	Mid-treatment	Gradual improvement	Alignment phase	Moderate	—	Adaptation and visible dental changes
	Post-treatment	Highest QoL improvement	After debonding/end of aligner therapy	High	Highest	Functional and esthetic gains; improved social confidence
Appliance Type and QoL Impact	Fixed appliances	—	Active treatment	Low	High	Highest early discomfort; reliable biomechanics
	Clear aligners	—	Active treatment	High	High	Best comfort and esthetics; dependent on patient compliance
	Lingual appliances	—	Active treatment	Moderate	High	Esthetically invisible; speech adaptation required

## Data Availability

No new data were created or analyzed in this study. Data sharing is not applicable to this article.

## Abbreviations

The following abbreviations are used in this manuscript:

OHRQoL	Oral Health-Related Quality of Life
OHIP	Oral Health Impact Profile
CPQ	Child Perceptions Questionnaire.
OIDP	Oral Impacts on Daily Performances
PIDAQ	Psychosocial Impact of Dental Aesthetics Questionnaire
